# Adherence to the new policy framework of the World Cancer Research Fund International in developing a policy package for the prevention of gastrointestinal cancers in Iran: a Delphi study

**DOI:** 10.1080/16549716.2021.1978661

**Published:** 2021-09-29

**Authors:** Ali Janati, Rahim Khodayari-zarnaq, Ahmad Khanijahani, Manouchehr Khoshbaten, Alireza Ghamkhar, Neda Kabiri

**Affiliations:** a Iranian Center of Excellence in Health Management, Department of Health Policy and Management, School of Management and Medical Informatics, Tabriz University of Medical Sciences, Tabriz, Iran; b Department of Health Policy and Management, School of Management and Medical Informatics, Tabriz Health Services Management Research Center, Tabriz University of Medical Sciences, Tabriz, Iran; c Department of Health Administration and Public Health, John G. Rangos School of Health Sciences, Duquesne University, Pittsburgh, PA, USA; d Liver and Gastrointestinal Disease Research Center, Tabriz University of Medical Sciences, Tabriz, Iran; e School of Nursing and Allied Medical Sciences, Maragheh University of Medical Sciences, Maragheh, Iran; f Research Center for Evidence based Medicine, Tabriz University of Medical Sciences, Tabriz, Iran; g Research Center of Psychiatry and Behavioral Sciences, Tabriz University of Medical Sciences, Tabriz, Iran

**Keywords:** Policy research, policy package, gastrointestinal cancer, prevention, Delphi survey

## Abstract

**Background:**

Gastrointestinal cancers in Iran are among the major non-communicable diseases with a considerable burden on the health system. Changes in lifestyles as well as environmental factors have resulted in the emergence of these cancers.

**Objective:**

To elicit and quantitatively verify experts’ opinions regarding the potential public health impact, feasibility, economic impact, and budgetary impact of gastrointestinal cancer prevention policies in Iran.

**Methods:**

Sixteen experts from Iran were recruited in an email-based, two-round Delphi study. In each round, a questionnaire of policy options for preventing gastrointestinal cancers, which adhered to the new policy framework of the World Cancer Research Fund International, was given to participants. In the first round, experts were asked to provide opinions for and against the policy options. The second round evaluated the policy options for their public health impact, feasibility, economic impact, and budgetary impact.

**Results:**

A total of 32 policy options were organized based on three domains: health-enhancing environments, system changes, and behavior change communications. Of the 32 policy options, there were consensus in 31 (96%) and 30 (93%) options for public health impact and feasibility, respectively. On study completion, experts reached a consensus in 29 of 32 (90%) policy options for economic impact; only on 26 (81%) of these policy options did participants reached consensus for budgetary impact.

**Conclusion:**

Findings indicated that although nearly all policy options reached a consensus for their public health impact, some options are not feasible or do not appear to have an economic rationale for being implemented. Moreover, it is crucial to take into account the inter-sectoral collaboration between health and non-health sectors. Findings from this study can be helpful for health policymakers in identifying support for evidence-informed approaches regarding gastrointestinal cancer prevention.

## Background

Non-Communicable Diseases (NCDs) are the leading cause of death worldwide which account for about 71% of the annual mortality, according to the World Health Organization (WHO) [1]. Controlling the major risk factors of NCDs, including unhealthy diet, low physical activity, tobacco/substance use, and alcohol abuse, can play a significant role in preventing and managing NCDs [2]. Four major NCDs with the top mortality rates include cardiovascular diseases, respiratory diseases, diabetes, and cancers [3].

Gastrointestinal cancers (GIC), as one of the deadliest cancers, accounted for 3.4 million deaths worldwide in 2018. Of the five major types of GIC, esophageal, gastric, and liver cancers are most prevalent in Asia. In contrast, pancreatic and colorectal cancers are most prevalent in Europe and North America [4]. A shift to Western lifestyle is identified as the primary factor responsible for the emergence and increase of the GIC in Asian countries, including Iran [5]. Additionally, often neglected environmental factors, including air and water pollution, have been shown to increase the risk of developing GIC [6]. Quite unfairly, groups of low socioeconomic backgrounds are more likely to be impacted by GIC due to lower education and limited access to preventive care, such as GIC screening and follow-up of abnormal test results [7].

Fortunately, primordial prevention and primary prevention can lessen the adverse effects and decrease the economic burden of GIC. Addressing lifestyle choices, the 66^th^ World Health Assembly endorsed the ‘WHO global action plan for the prevention and control of NCDs 2013–2020’, with nine global targets, including a 25% relative reduction in premature mortality from NCDs by 2025 [8]. A recent study used the OECD’s Strategic Public Health Planning for NCDs to estimate the health and economic impact of six primary prevention interventions on the major risk factors of cancer, including unhealthy diet, physical inactivity, and alcohol abuse. The results showed that these interventions led to a decrease in new cancer cases, reduction in costs, and improvements in the financial sustainability of the health system [9]. Another study, conducted in Mexico, assessed the impact of primary prevention policies such as improving healthy diet and increasing physical activity to prevent NCDs. Findings indicated that primary prevention policies successfully prevented NCDs in low and middle-income countries [10].

Most of the previously developed GIC prevention programs only consider the health sector effects in the implementation phase. However, due to the wide variety of risk factors, GIC prevention is a multifaceted topic that requires a comprehensive approach in all policymaking stages. It is crucial to improve public health outcomes and achieve an effective policy design and translation. An example is a multi-sectoral partnerships program which included local non-governmental organizations (NGOs), community health centers, and public health agencies implemented in Los Angeles to increase colorectal cancer screening rates [11].

## Methods

This two-round policy Delphi study aimed to inform evidence-based policymaking for GIC prevention in Iran by systematically analyzing experts’ opinions [12]. Experts were asked to rate the potential public health impact, feasibility, economic impact, and budgetary impact of several GIC prevention policies compiled from two previous studies. The policy Delphi method allows researchers to improve their opinions based on the views of other experts during rounds that can be continued until a consensus is reached [13]. The goal of a policy Delphi method was to identify all supporting and contrasting perspectives on GIC prevention policies. In contrast to conventional Delphi, policy Delphi is conducted to reach a conclusion based on consensus [14,15].

### Survey design

Policy options were constructed based on the results of two previous studies conducted by the authors. The first study extracted GIC prevention policies through a qualitative systematic review and meta-synthesis of existing programs and strategies. From this study, 12 categories were identified. In the second study (a qualitative framework-based study) [16], GIC prevention policies in Iran were analyzed through semi-structured interviews and the analysis of national documents using a policy triangle framework. A total of seven themes and 123 sub-themes were extracted through two studies resulting in 25 policy options (Table 1).

**Table 1.** Predetermined policy options used in round 1 of the Delphi survey

Policy options
Controlling and monitoring food processing and food distribution industries
Controlling and monitoring food imports
Encouraging physical activity in the society and equipping parks with sports facilities and exercise equipment
Allocating more funds to gastrointestinal cancer prevention and screening programs
Higher taxation on unhealthy food items
Allocating targeted subsidies on healthy foods (such as fruits and vegetables) for the lower socioeconomic groups
Restricting the marketing of unhealthy foods
Restricting the marketing of unhealthy lifestyle behaviors
Restricting opium availability and accessibility
Prohibiting harmful pesticides in the agriculture section
Promoting safe food products and production practices
Limiting the availability of unhealthy fast foods for the young population
Promoting healthy transportation policies
Promoting safe water supply policies
Improving multi-sectoral engagement across government sectors in gastrointestinal cancer prevention policy process
Improving the fair distribution of GIC diagnostic devices and gastroenterologists in the secondary care
Promoting the collaboration between NGOs and the private sector in financing and service provision of related activities in the secondary care
Encouraging NGOs and the scientific community to participate in the policymaking process
Establishing public education campaigns on healthy lifestyles and gastrointestinal cancers prevention and screening
Promoting efficiency in primary health care (e.g. delivering Fecal-Immunochemical Test by mail to all target populations)
Promoting oral health services
Helicobacter pylori screening for individuals under 40 years old
Integrating physical activity promotion programs in primary health care services
Giving more priority to evidence-based screening programs in strategic purchasing of services
Educating and Enhancing knowledge and awareness of health managers and policymakers at different levels of the decision-making process

**Figure 1.**
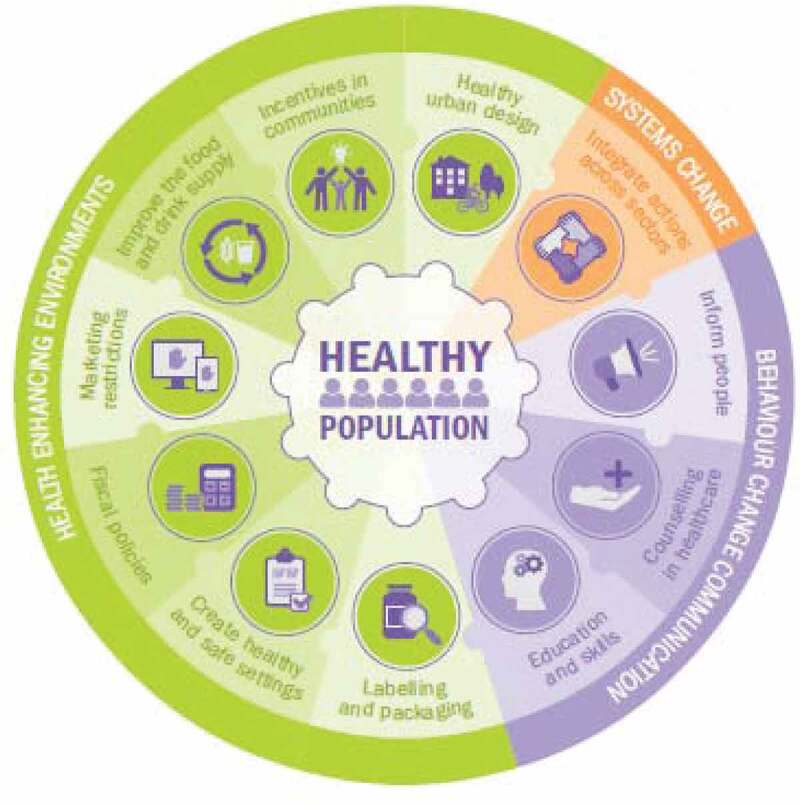
The new policy framework outlined by the World Cancer Research Fund (WCRF) International [17]

The predetermined policy options were then organized using the new policy framework developed by the World Cancer Research Fund (WCRF) International [17]. This framework brings together policy options in three domains of health-enhancing environments, system changes, and behavior change communication. These three domains include the following 11 policy areas: labeling and packaging, creating healthy and safe settings, fiscal policies, marketing restrictions, improving the food and beverage supply, incentives for communities, healthy urban designs, integrating actions across sections, informing people, counseling in healthcare, education, and skills (Figure 1).

### Questionnaire design

Twenty-five policy options were included in the first-round questionnaire. Participants were asked to choose whether they were for or against each policy. Additionally, an open text box was provided for each policy option for any additional comments and explanations. The results from the first round were used to revise the policy options (i.e. a few options were eliminated or divided into separate options, resulting in 32 final policy options).

The second-round questionnaire included 32 policy options. Analyzing and prioritizing policy options was guided by CDC’s (Centers for Disease Control and Prevention) policy analytical framework. This framework provides a method for policymakers to consider policies that can improve health while considering economic impact [18]. The CDC’s policy analytical framework offers four criteria for analyzing policy options: public health impact, feasibility, economic impact, and budgetary impact. Scoring for public health impact and feasibility was ranked as ‘low’, ‘medium’, or ‘high’. While, for clarity, the economic and budgetary impact descriptors were ‘less favorable’, ‘favorable’, and ‘more favorable’ in the CDC’s policy analytical framework. For simplicity, we quantified the rankings on a 3-point scale. Also, an empty column was provided within the questionnaire to identify the agencies responsible for or supporting the policy options implementation at the national level.

### Delphi process

For both rounds, questionnaires with a cover letter explaining the study background and scoring criteria were sent via email. The first round of Delphi was conducted in July and August 2020, and the second round was conducted in September 2020. A maximum of two reminders was sent per round in cases of no response after two weeks.

### Study participants

A total of 53 participants in the health sector and non-health sector, including academics and policymakers who were active in the field of GIC policymaking, were contacted and invited to the first Delphi round. The sample size of experts in a Delphi study, depending on the policy issue area, the complexity of the study, and resources, usually range from 15 to 30 participants [19]. Scientific journal publications and media interviews were used to identify the first group of participants, followed by a snowball sampling method to recruit additional participants. Only those who completed the first-round questionnaire were subsequently invited to participate in the second round of the Delphi study. Further details regarding the characteristics of participants are provided in the results section.

### Data analysis

Analysis of the responses from the two rounds of the Delphi study was performed immediately after completing the questionnaires. First, a thematic analysis [20] of the comments on policy options was conducted at the end of the first Delphi round, resulting in omission or rephrasing of the policy options as required. Subsequently, the consensus was determined to be ‘met’ if 70% or more participants selected ‘high’, ‘medium’, ‘more favorable’, and ‘favorable’ for a given policy option. Otherwise, the consensus was considered as ‘not met’. The criteria for consensus were determined before the start of the study by members of the research team. Furthermore, the results of the selected responsible national and governmental agencies were analyzed by content analysis [20], based on the repetition of the identified organizations.

## Results

### Expert panel

Of the 53 invited experts, 16 participated in the first round of the Delphi (response rate 30%), and 15 experts responded to the second questionnaire (response rate: 93%). The main reasons for non-response were lack of time and the necessary expertise in completing the questionnaire. The main characteristics and professional backgrounds of participants are presented in Table 2.

**Table 2.** Characteristics of the Delphi expert panel

Expert	Gender	Professional background	Completed 2^nd^ round (Yes/No)
1	M	Health economist	Y
2	F	Public economist	Y
3	M	Medical specialist/gastroenterologist	Y
4	M	Health policy/researcher	Y
5	M	Health policy/academic	Y
6	M	Health education and promotion	Y
7	M	Health policy/academic	Y
8	M	Health policy/researcher	Y
9	M	Community medicine/futures studies in health	Y
10	F	Health policy/researcher	Y
11	M	Health services management/non-communicable diseases	Y
12	F	Health policy/researcher	N
13	M	Medical specialist/gastroenterologist	Y
14	M	Health services management/non-communicable diseases	Y
15	M	Health policy/academic	Y
16	M	Epidemiologist	Y

### Round 1

Several revisions were made to the policy options based on the thematic analysis of the open comments in the first round of Delphi. Two options were merged to form a comprehensive concept (i.e. ‘Improving the fair distribution of GIC diagnostic devices and gastroenterologists in the secondary care’ and ‘Promoting the collaboration between Non-Governmental Organizations (NGOs) and the private sector to participate in financing and service provisionof related activities in the secondary care’). Furthermore, several policy options were rephrased or broken down into more specific pieces to increase precision and comprehensiveness. For instance, participants proposed changing ‘promoting healthy transportation policies’, which implicitly emphasized the promotion of clean air, to three options of ‘promoting clean air policies’, ‘promoting transportation policies that encourage walking, cycling, and use of public transportation’, and ‘establishing a safe environment for walking and cycling in urban areas’. As such, ‘establishing public education campaigns on healthy lifestyles and gastrointestinal cancers prevention and screening’ was divided into multiple options of ‘establishing public education campaigns on healthy lifestyle’, ‘establishing public education campaigns on gastrointestinal cancers prevention and screening’, ‘prohibiting the promotion of inactive lifestyles through media, especially for children and adolescents’, and ‘including content related to the consumption of healthy foods in the educational curriculum of schools’.

### Round 2

Revising the policy options based on the expert’s comments resulted in 32 policy options included for the second round. The level of agreement for the criteria of the second round is presented in Table 3. All policy options reached a consensus for overall health impact except ‘educating and enhancing knowledge and awareness of health managers and policymakers in different levels of the decision-making process’. Two options did not reach a consensus in terms of feasibility (i.e. ‘restricting the availability and accessibility of opium’ and ‘giving more priority to evidence-based screening programs in strategic purchasing of services’). In addition, the second round results indicated that some policy options, while feasible and likely to have a tremendous public health impact, may not have an economic rationale. One instance is ‘helicobacter pylori (h-pylori) screening for individuals less than 40 years old’.

**Table 3.** Round 2 Delphi survey results of the four criteria for policy options

Policy options	Level of agreement			
	Public health impact	Feasibility	Economic impact (Cost-effectiveness)	Budgetary impact
Controlling and monitoring food processing industries	93%	80%	100%	93%
Controlling and monitoring food distribution centers	93%	80%	100%	93%
Controlling and monitoring food imports	87%	80%	87%	87%
Encouraging physical activity in schools, universities, and workplaces	100%	93%	100%	87%
Allocating more funds to gastrointestinal cancer prevention and screening programs	100%	73%	93%	93%
Higher taxation on unhealthy food items	93%	73%	93%	87%
Allocating targeted subsidies on healthy foods (such as fruits and vegetables) for the lower socioeconomic groups	100%	80%	93%	93%
Restricting the marketing of unhealthy foods	100%	73%	100%	93%
Restricting the marketing of unhealthy eating behaviors	100%	73%	100%	93%
Prohibiting the promotion of inactive lifestyles through media, especially for children and adolescents	100%	80%	100%	93%
Restricting opium availability and accessibility	100%	40%	87%	87%
Prohibiting harmful pesticides in the agriculture section	100%	73%	80%	73%
Promoting safe food products and production practices	100%	93%	100%	87%
Limiting the availability of unhealthy fast foods for the young population	100%	73%	83%	87%
Making a safe environment for walking and cycling in urban areas	87%	87%	100%	53%
Equipping parks with sports facilities and exercise equipment	100%	93%	100%	87%
Promoting transportation policies that encourage walking, cycling, and use of public transportation	87%	87%	100%	53%
Promoting safe water supply policies	93%	93%	93%	93%
Promoting clean air policies	87%	87%	100%	53%
Improving multi-sectoral engagement across government sectors in gastrointestinal cancer prevention policy process	100%	93%	93%	80%
Making fair distribution of gastroenterologists in rural Iran with the participation of the private sector and Non-Governmental Organizations (NGOs)	80%	73%	93%	87%
Making fair distribution of gastrointestinal cancer screening and diagnostic devices (e.g. colonoscopy and endoscopy) in rural Iran with the participation of the private sector and NGOs	80%	73%	93%	87%
Encouraging NGOs and the scientific community in the policymaking process	93%	87%	93%	93%
Establishing public education campaigns on healthy lifestyles	87%	73%	67%	53%
Establishing public education campaigns on gastrointestinal cancers prevention and screening	87%	73%	67%	53%
Promoting efficiency in primary health care (e.g. delivering Fecal-Immunochemical Test* by mail for all target populations).	100%	87%	100%	93%
Promoting active care provision in oral health**	87%	87%	100%	93%
Helicobacter pylori screening for individuals under 40 year-olds***	87%	80%	67%	60%
Integrating physical activity promotion programs in primary health care services	93%	87%	100%	100%
Giving more priority to evidence-based screening programs in strategic purchasing of services	100%	67%	100%	93%
Educating and enhancing knowledge and awareness of health managers and policymakers at different levels of the decision-making process	67%	80%	80%	80%
Including content related to the consumption of healthy foods in the educational curriculum of schools	100%	87%	100%	80%

*: Disease-specific policy option for the screening of colorectal cancer

**: Disease-specific policy option for the prevention of esophageal cancer

***: Disease-specific policy option for the screening of gastric adenocarcinoma (non-cardia)

### Final policy options

A list of all 32 policy options organized based on the WCRF International new policy framework, along with the national agencies responsible for implementing each option, are presented in Table 4. Seven policy areas and 19 policy actions were associated with the domain of ‘health-enhancing environment’. These policy options aim to promote an individual’s healthy behaviors and focused on environmental factors related to GIC. Only one policy area and four policy options were included in ‘system changes’, focusing on the engagement of different governmental health and non-health sectors in GIC prevention and the private sector and NGOs. Three policy areas and nine policy options were associated with the final domain of ‘behavior change communication’, highlighting the role of improving awareness of the population, policymakers, and health managers regarding GIC. Moreover, disease-specific screening programs for GIC are included in this domain.

**Table 4.** Policy framework for a package of actions needed to prevent gastrointestinal cancers

Domains	Policy areas	Policy options	The national focal point for implementation
Health-enhancing environments	Labeling and packaging	Controlling and monitoring food processing industries	Government, Health Commission in the Parliament of Iran, and the Supreme Council of Health and Food Security
		Controlling and monitoring food distribution centers	General Inspection Organization, Standard Organization
		Controlling and monitoring food imports	Ministry of Internal Affairs, Ministry of Industry, Mine, and Trade
	Create healthy and safe settings	Encouraging physical activities in schools, universities, and workplaces	Ministry of Sport and Youth, Ministry of Education, Ministry of Welfare, Labor and Social Affairs
	Fiscal policies	Allocating more funds to gastrointestinal cancer prevention and screening programs	Ministry of Health
		Higher taxation on unhealthy food items	Parliament of Iran, The Cabinet
		Allocating targeted subsidies (such as fruits and vegetables) on healthy foods for the lower socioeconomic groups	Parliament of Iran, The Cabinet, Organization of Targeted Subsidies
	Marketing restrictions	Restricting the marketing of unhealthy foods	Parliament of Iran, Ministry of Industry, Mine, and Trade, Islamic Republic of Iran Broadcasting, Municipalities
		Restricting the marketing of unhealthy eating behaviors	Parliament of Iran, Islamic Republic of Iran Broadcasting, Municipalities
		Prohibiting the promotion of inactive lifestyles through media, especially for children and adolescents	Parliament of Iran, Islamic Republic of Iran Broadcasting
		Restricting opium availability and accessibility	Police force (Iran Drug Control Headquarters)
	Improve the food and drink supply	Prohibiting harmful pesticides in the agriculture section	Ministry of Agriculture, Standard Organization
		Promoting safe food products and production practices	General Inspection Organization, Standard Organization
		Limiting the availability of unhealthy fast foods for the young population	General Inspection Organization, Standard Organization
	Incentives in communities	Making a safe environment for walking and cycling in urban areas	Municipalities
		Equipping parks with sports facilities and exercise equipment	Municipalities, Ministry of Sport and Youth
	Healthy urban designs	Promoting transportation policies that encourage walking, cycling, and use of public transportation	Ministry of Road and Transportation, Road Maintenance and Transportation Organization, municipalities
		Promoting safe water supply policies	Ministry of Energy
		Promoting clean air policies	Organization of environment, Ministry of Road and Transportation, Road Maintenance and Transportation Organization, Municipalities
System change	Integrate actions across sectors	Improving multi-sectoral engagement across government sectors in gastrointestinal cancer prevention policy process	Health Commission in the Parliament of Iran, the Supreme Council of Health and Food Security
		Making fair distribution of gastroenterologists in rural Iran with the participation of private sector and Non-Governmental Organizations (NGOs)	Ministry of Health, The Cabinet
		Making fair distribution of gastrointestinal cancer screening and diagnostic devices (e.g. colonoscopy and endoscopy) in rural Iran with the participation of the private sector and NGOs	Ministry of Health, The Cabinet
		Encouraging NGOs and the scientific community to participate in the policymaking process	Ministry of Health
Behavior change communication	Inform people	Establishing public education campaigns on healthy lifestyles	Ministry of Health
		Establishing public education campaigns on gastrointestinal cancers prevention and screening	Ministry of Health
	Counseling in healthcare	Promoting efficiency in primary health care (e.g. delivering Fecal-Immunochemical Test by mail for all target populations).	Ministry of Health
		Promoting active care provision in oral health	Ministry of Health
		Helicobacter pylori screening for individuals under 40 years old	Ministry of Health
		Integrating physical activity promotion programs in primary health care services	Ministry of Health
		Giving more priority to evidence-based screening programs in strategic purchasing of services	Ministry of Health
	Education and skills	Educating and enhancing knowledge and awareness of health managers and policymakers at different levels of the decision-making process	Ministry of Health
		Including content related to the consumption of healthy foods in the educational curriculum of schools	Health Commission in the Parliament of Iran, Ministry of Health, Ministry of Education

Data analysis of responsible national agencies for policy options indicated that a wide range of non-health sector organizations and the Ministry of Health (MoH) are responsible for implementing GIC prevention policy options.

## Discussion

This study aimed to quantitatively verify policy options for GIC prevention in Iran using a Delphi approach. This approach enabled a practical assessment of GIC prevention policy options by purposively recruited experts. To our knowledge, this is the first study that attempts to provide a comprehensive package of policies to prevent GIC in Iran and the opinions of an expert panel on this issue. In this study, we provided a panel of experts with a questionnaire of 32 policy options for GIC prevention, requesting them to rate the four criteria of public health impact, feasibility, economic impact, and budgetary impact. The final analysis of our data on the public health impact of policy options showed that consensus was reached in all policy options except one. This indicates that the policy options were appropriately designed. Moreover, of the 32 policy options, there were consensus in 31 (96%), 29 (90%), and 26 (81%) policy options for feasibility, economic impact, and budgetary impact, respectively.

Population-based programs for GIC screening are recognized as a significant public health intervention by policymakers across the world. Overall, these programs are disease-specific and primarily target early screening interventions for cancers. For instance, screening programs for colorectal cancer consider fecal immunochemical test (FIT) as an initial screening test followed by a diagnostic colonoscopy for those with a positive FIT result [21,22]. Besides, there has been a rise in primary prevention programs aiming to modify the existing risk factors to prevent diseases. These programs focus on promoting healthy dietary habits, physical activity, and reducing smoking and alcohol consumption [23,24]. Our results included disease-specific policies (e.g. FIT for colorectal cancer screening and helicobacter pylori screening for gastric cancer) along with the primary prevention policies.

All three policy options reached a consensus in the policy area of labeling and packaging of the current policy package. In Fiji, economic and agricultural policy changes and country membership in the World Trade Organization lead to systematic monitoring of imported food. Consequently, the consumption of healthy foods such as fresh fruits and vegetables and whole-grain refined cereals increased [25]. A study implementing a new nutrition-labeling scheme for children indicated that the scheme discouraged 6–12 years old children from consuming products attributed to NCDs [26].

In the policy area of creating healthy and safe settings and infrastructures, we suggested encouraging physical activities in schools, universities, and workplaces. Similarly, building healthy and safe community environments in early adulthood appears to reduce exposure to cancer-related risk factors. Balancing the contextual factors beyond the physical environment -such as marketing unhealthy food products- has also been shown to impact young adults’ eating behaviors [27]. In one study, Marketing unhealthy food in smartphone applications, social media, and other websites targeting Australian children under 13 years old promoted the unhealthy food consumption habits in this group [28]. The WCRF International developed a policy package to promote healthy diets and reduce obesity and other diet-related NCDs. The framework is called NOURISHING which each letter represents policy areas in the subject [24]. The unmet consensus on the feasibility of restricting the availability and accessibility of opium is essential to consider. The high prevalence of opium abuse in Iran, especially among young people, can mainly be traced back to the country’s history of opium production [29].

Fiscal policies such as taxing and subsidizing food items were suggested in the current study as a policy option for preventing gastrointestinal cancers, which reached a consensus of Delphi participants in the four aspects. Similarly, subsidies on healthy products in the USA had a high impact and feasibility in reducing the cancer burden [30]. A literature review of New Zealand studies indicated that taxes and subsidies on food/beverage had favorable health impacts in controlling NCDs [31].

The next policy area – improving the food supply- included policy options for promoting healthy and safe food products and production practices. Findings of a study on the use of home yards in supporting food security programs in rural areas of Bangli revealed that consumption of food groups of vegetables and fruits exceeds the desired level of consumption [32]. Adhering to the policy framework by the World Cancer Research Fund International, consumption of healthy foods was increased. In contrast, as expected, the consumption of fast foods and other unhealthy food products was decreased in Sweden [33].

Despite the general views in favor of clean air through cycling and walking, most experts in the current study acknowledged that promoting clean air policies through establishing a safe environment for walking and cycling in urban areas is costly and needs substantial monetary investment. A plan to promote daily bicycle use in Tehran was estimated to cost €40 million, with the final objective of improving bicycle use from 7% to 12% by 2030. The involvement of private sector investment in renting bicycles and other sports facilities and equipment seems to be helpful [34]. Cycling infrastructures, such as exclusive continuous bikeways, high-quality road surfaces, and bicycle parking spaces, need an enormous budget in low and middle-income countries, raising concerns about the cost-effectiveness of such intervention [35].

Multi-sectoral engagement of governmental and non-governmental organizations and expansion of the private and non-governmental sector in the service delivery is recommended in the current policy package to prevent gastrointestinal cancers. The logic behind this policy option was to improve equity in access to cancer screening and diagnostic devices in rural and disadvantaged areas. The evidence from Nepal and Bangladesh indicated that expansion of service delivery by the private sector narrowed the gaps between the wealthy and the poor in access to necessary care [36].

In contrast to the increasing number of health awareness campaigns worldwide and their impact on the screening of GIC [37], the expert panel did not reach a consensus on the cost-effectiveness of these campaigns. Based on the experts’ opinions, this weak consensus is mainly because of awareness campaigns’ unstable and narrow focus. Theme-day campaigns were found similarly to be less important by the expert panel to prevent and control NCDs in China [38]. The results of a review article reported similar challenges. Health awareness campaigns targeting alcohol consumption, despite the low cost, did not notably affect consumption levels or health outcomes and were not cost-effective [39]. By contrast, a literature review showed the high cost-effectiveness of awareness campaigns aimed to reduce sodium intake [40]. Additionally, smoking cessation campaigns in London were more cost-effective when targeting high-risk populations than the general population [41]. As a result, it is better to employ targeted awareness campaigns on high-risk groups (e.g. the elderly with at least one type of chronic disease or patients who benefit from such interventions) rather than one-size-fits-all population-wide interventions.

As apparent from the experts’ comments, h-pylori screening of the under-40-years-old population may not be implemented due to economic considerations, while essential and likely to influence public health. A randomized controlled trial conducted in Denmark, a country with an h-pylori prevalence of 17.5%, indicated that h-pylori screening of the population was not cost-effective, nor did these interventions improve quality of life [42]. On the contrary, h-pylori screening of high-risk populations in an occupational health setting in Japan, where the prevalence of gastric cancer and h-pylori is high [43], has shown to be cost-effective [44]. Similarly, findings from a study conducted in New Zealand, a high-income country with an age-standardized incidence of 8 per 100,000 for gastric cancer, indicated that the cost-effectiveness of h-pylori screening was more favorable for a high-risk group than the general population [45]. As evident, a greater probability of cost-effectiveness of h-pylori screening can be expected in contexts with a high prevalence of h-pylori. A meta-analysis showed that the prevalence of h-pylori infection among the Iranian population was 54% [46]. Despite the high prevalence of h-pylori in Iran, many factors should be considered in implementing h-pylori screening programs. Implementing h-pylori screening programs without considering the country’s economic status can worsen health inequalities. Therefore, policymakers should consider the burden of gastric cancer, other health priorities, and comparative cost-effectiveness analysis when planning to implement population-based h-pylori screening programs [47].

This study also demonstrated significant public health impacts and economic rationale for evidence-based guidelines on screening programs in strategic purchasing of services. However, this policy option does not appear to be feasible, mainly due to challenges attributable to strategic purchasing infrastructures [48,49]. This finding can prompt policymakers to consider the creation of infrastructures to implement this method. Moreover, guideline adherence of health services providers, particularly regarding high-cost screening services, should be enforced to avoid unnecessary costs to patients, insurance companies, and the health system.

The last policy option in the framework -including content related to the consumption of healthy foods in the educational curriculum of schools- is shown to be an effective option in similar studies. For example, developing a cancer risk-reduction education tool focusing on the wide range of cancers for high school and college students in New York City is shown to be a successful program [50]. Similarly, pilot implementation of a cancer education intervention for middle and high school students in Kentucky increased the cancer literacy level among the target population [51].

A wide range of factors influences the health of individuals. One major characteristic of NCDs, including cancers, is that they are, to some extent, affected by environmental factors not directly related to health. These wicked problems require a comprehensive policymaking process regarding policy engagement of non-health sectors whose primary concern is not health. This holistic approach will improve the accountability of public policymakers for considering health implications in making policies [52]. The expert panel in this study identified many non-health sector organizations. However, the roles and responsibilities of these organizations in GIC prevention should be clarified for successful policy implementation.

### Strengths and limitations

This study included policy options for the prevention of GIC in Iran and the opinions of a group of experts about the public health impacts, feasibility, and economic and budgetary impacts of these policies. GIC shares many common risk factors with other cancers and various NCDs. The proposed policies in this study could have benefits beyond GIC prevention. One other strength of this study is that the generalizability of policy options. These policy options were mainly built based on a qualitative systematic review that included programs and strategies worldwide. Apart from the feasibility and financial considerations that depend on cultural, economic, political, and health system contexts, we expect that the implementation of this policy package is generalizable to other similar healthcare settings and nations.

The small sample size was one limitation of this study. The research team’s conscious decision was to include qualified experts than a broad spectrum of participants. The consistent, in-depth comments received in the first Delphi round indicated the sufficiency of the sample size. However, given that several policy options in the package are related to set rules and regulations, it could be seen as a limitation that this study did not include experts from the country’s legislative body. A low response rate was another limitation of this study. One of the compelling reasons for the low response rate was that this Delphi study was conducted during the novel coronavirus diseases (COVID-19) pandemic. During this time, most experts were busy carrying out their specialized duties and challenges in this pandemic. Furthermore, most of the invited panelists who did not participate in the study indicated their lack of expertise in cancer prevention. Experts were invited from different governmental agencies such as the Ministry of Sports and Youth; the Ministry of Industry, Mine, and Trade; the Ministry of Agriculture; the Ministry of Education, and the Islamic Republic of Iran Broadcasting.

## Conclusion

This study identified 32 policy options categorized under three domains and 11 policy areas. Domains of the policy package include health-enhancing environments, system changes, and behavior change communication. It is crucial to create a shared vision and shared policy goals between health and non-health sectors for these policy options to be successfully designed and implemented. More importantly, expert opinions showed that considering all policy options might not be feasible or economically rational to implement even though having good public health impact. The implementation of some policies might shift scarce resources away from efficient prevention programs. Findings from this study may be helpful for health policymakers in identifying support for evidence-informed approaches regarding GIC. As a next step, the translation of these policy options into the implementation phase should be considered. Furthermore, economic evaluations should be performed for potentially low cost-effective policy options.

## Paper context

Gastrointestinal cancers are one of the deadliest cancers in the world. The current study developed a multifaceted policy package containing 32 policy options to prevent gastrointestinal cancers. Policymakers in Iran and contextually similar countries may use this policy package in preventing these cancers.
